# Building gender-specific sexually transmitted infection risk prediction models using CatBoost algorithm and NHANES data

**DOI:** 10.1186/s12911-024-02426-1

**Published:** 2024-01-24

**Authors:** Mengjie Hu, Han Peng, Xuan Zhang, Lefeng Wang, Jingjing Ren

**Affiliations:** 1 Department of General Practice, First Affiliated Hospital, Zhejiang University School of Medicine, 310003 Hangzhou, China; 2 Clinical Research Institute, Zhejiang Provincial People’s Hospital (Affiliated People’s Hospital of Hangzhou Medical College), Hangzhou, China; 3 Department of Cardiology, The First Affiliated Hospital, Zhejiang University School of Medicine, 310003 Hangzhou, China; 4 Kidney Disease Center, the First Affiliated Hospital, College of Medicine, Zhejiang University, 310003 Hangzhou, China

**Keywords:** Sexually transmitted infections, CatBoost algorithm, NHANES data, SHAP algorithm

## Abstract

**Background and aims:**

Sexually transmitted infections (STIs) are a significant global public health challenge due to their high incidence rate and potential for severe consequences when early intervention is neglected. Research shows an upward trend in absolute cases and DALY numbers of STIs, with syphilis, chlamydia, trichomoniasis, and genital herpes exhibiting an increasing trend in age-standardized rate (ASR) from 2010 to 2019. Machine learning (ML) presents significant advantages in disease prediction, with several studies exploring its potential for STI prediction. The objective of this study is to build males-based and females-based STI risk prediction models based on the CatBoost algorithm using data from the National Health and Nutrition Examination Survey (NHANES) for training and validation, with sub-group analysis performed on each STI. The female sub-group also includes human papilloma virus (HPV) infection.

**Methods:**

The study utilized data from the National Health and Nutrition Examination Survey (NHANES) program to build males-based and females-based STI risk prediction models using the CatBoost algorithm. Data was collected from 12,053 participants aged 18 to 59 years old, with general demographic characteristics and sexual behavior questionnaire responses included as features. The Adaptive Synthetic Sampling Approach (ADASYN) algorithm was used to address data imbalance, and 15 machine learning algorithms were evaluated before ultimately selecting the CatBoost algorithm. The SHAP method was employed to enhance interpretability by identifying feature importance in the model’s STIs risk prediction.

**Results:**

The CatBoost classifier achieved AUC values of 0.9995, 0.9948, 0.9923, and 0.9996 and 0.9769 for predicting chlamydia, genital herpes, genital warts, gonorrhea, and overall STIs infections among males. The CatBoost classifier achieved AUC values of 0.9971, 0.972, 0.9765, 1, 0.9485 and 0.8819 for predicting chlamydia, genital herpes, genital warts, gonorrhea, HPV and overall STIs infections among females. The characteristics of having sex with new partner/year, times having sex without condom/year, and the number of female vaginal sex partners/lifetime have been identified as the top three significant predictors for the overall risk of male STIs. Similarly, ever having anal sex with a man, age and the number of male vaginal sex partners/lifetime have been identified as the top three significant predictors for the overall risk of female STIs.

**Conclusions:**

This study demonstrated the effectiveness of the CatBoost classifier in predicting STI risks among both male and female populations. The SHAP algorithm revealed key predictors for each infection, highlighting consistent demographic characteristics and sexual behaviors across different STIs. These insights can guide targeted prevention strategies and interventions to alleviate the impact of STIs on public health.

**Supplementary Information:**

The online version contains supplementary material available at 10.1186/s12911-024-02426-1.

## Introduction

Sexually transmitted infections (STIs) pose a significant global public health challenge due to their high incidence rates, which exert substantial pressure on both family and national healthcare budgets while concurrently impairing individual quality of life [1, 2]. Moreover, the widespread issue of delayed STI diagnosis raises the risk of severe consequences such as compromised reproductive and neonatal health when early intervention is neglected [3]. Research indicates an upward trend in both absolute cases and disability-adjusted life years (DALYs) for STIs between 1990 and 2019 [4]. Syphilis, chlamydia, trichomoniasis, and genital herpes have demonstrated an increasing trend in age-standardized rates (ASRs) from 2010 to 2019 [5]. Consequently, STIs remain a persistent global public health concern. Furthermore, since 2010, the age-standardized incidence rate among young people has exhibited an upward trend, particularly regarding syphilis [4]. As such, early intervention through STI prediction is crucial [6].

Machine learning (ML) offers significant advantages in disease prediction, with numerous studies already exploring its potential for STI prediction. Bao et al. [7] aimed to develop and evaluate the performance of machine learning models in predicting the diagnosis of HIV and STIs based on a large retrospective cohort of Australian men who have sex with men (MSM). Fieggen et al. [8] discussed crucial considerations when selecting variables for model development and evaluating the performance of various machine learning algorithms, as well as the potential role of emerging tools such as Shapley Additive Explanations in understanding and decomposing these models in the context of HIV. Xu et al. [9] sought to identify determinants and predict chlamydia re-testing and re-infection within one year among heterosexuals with chlamydia to pinpoint potential PDPT (Patient-Delivered Partner Therapy) candidates.

Our study developed male-based and female-based STIs risk prediction models using the CatBoost algorithm, employing data from the National Health and Nutrition Examination Survey (NHANES) for training and validation. Sub-group analyses were conducted for each STI, including genital herpes, genital warts, gonorrhea, and chlamydia infections. The female sub-group also encompassed human papillomavirus (HPV) infection.

## Methods

### Data source

NHANES is a series of studies aimed at evaluating the health and nutritional status of adults and children in the United States [10]. As a significant initiative of the National Center for Health Statistics (NCHS), NHANES contributes to the Centers for Disease Control and Prevention’s (CDC) mission by generating essential health statistics for the nation.

Data were collected from the NHANES datasets spanning 2009 to 2016, encompassing 19,998 individuals aged between 18 and 59 years. The questionnaires from different years exhibited subtle variations. For example, beginning in 2015–2016, modifications on question wording and response categories were made to the sexual orientation question, specific to males and females. Initially, we reviewed 53 questions, but due to variations and relevance, the final selection included 48 questions that were consistent across all surveys. A total of 7,945 individuals were excluded due to their responses to the Sexual Behavior Questionnaire, specifically those who provided answers other than “yes” or “no” regarding whether a doctor had ever informed them of having HPV, genital herpes, genital warts, gonorrhea, or chlamydia, or those who refused to answer the questions. Consequently, the final sample comprised 12,053 participants, including 6,163 females and 5,890 males.

### Feature selection

The study incorporated general demographic characteristics (gender, age, education level, and marital status) along with questions from the Sexual Behavior Questionnaire (codes and corresponding questions are accessible on the NHANES website: https://wwwn.cdc.gov/nchs/nhanes). The feature selection process includes identifying the consistent questions in all NHANES versions. Since some questionnaire items targeted exclusively either the male or female population, and questions serving as labels were excluded, the analysis for the female population included 30 features, while that for the male population comprised 33 features. For missing data, we applied imputation methods tailored to the data type. In addition, to ensure the comparability of feature scales across different measures, we implemented a normalization process. Specifically, we utilized the Normalized Gini Coefficient, which scales data within a range from 0 (indicating perfect equality) to 1 (indicating maximum inequality). This normalization step is crucial in maintaining consistency and reliability in the comparative analysis of our dataset features.

#### Balance of data

To address data balance issues, we reviewed literature such as Johnson and Khoshgoftaar’s work on deep learning with class imbalance [11] and Majority Weighted Minority Oversampling Technique (MWMOTE) [12]. We chose not to use random under or oversampling due to potential data loss or overfitting. Instead, we utilized the Adaptive Synthetic Sampling Approach (ADASYN) [13], considering its effectiveness in managing imbalanced datasets.

### Algorithm

We carried out risk prediction modeling for various STIs cases within the study population using 15 unique machine learning algorithms, including Quadratic Discriminant Analysis, Extra Trees Classifier, Random Forest Classifier, Light Gradient Boosting Machine, CatBoost Classifier, Gradient Boosting Classifier, Ada Boost Classifier, Decision Tree Classifier, K Neighbors Classifier, Ridge Classifier, Linear Discriminant Analysis, Logistic Regression, SVM - Linear Kernel, Naive Bayes, Dummy Classifier. In evaluating the performance of our model, we employed a comprehensive set of metrics, including Accuracy, Area Under the Curve (AUC), Recall, Precision (Prec), F1 Score, Cohen’s Kappa, and Matthews Correlation Coefficient (MCC). After thoroughly evaluating and comparing the performance of these models, we ultimately chose the CatBoost algorithm.

The CatBoost algorithm is a robust and highly efficient gradient boosting framework extensively employed in machine learning applications [14]. It outperforms traditional gradient boosting techniques, especially when managing complex datasets featuring numerous categorical variables. The strength of the CatBoost algorithm lies in its capacity to handle feature interactions accurately while minimizing overfitting, thereby ensuring exceptional predictive power.

Python 3.12.0 was used to the balance of data. PyCaret 2.3.1 in Jupyter Notebook was used to train and validate the CatBoost classifier. The “compare_models()” and “create_model” functions in PyCaret were used, which automatically handles data preprocessing, and then train and evaluate multiple models using 10-fold cross-validation, streamlining the selection of the most effective model based on performance metrics.

### Interpretability

To enhance the interpretability of the CatBoost model, we employed the SHAP (SHapley Additive exPlanations) technique. This approach provides insights into how each feature contributes to the model’s prediction, allowing for a better understanding of the model’s decision-making process.

## Results

### Basic characteristics

Table 1 presents the demographic characteristics of the study participants. The mean age is approximately 39 years for both males (*n* = 5,890) and females (*n* = 6,163). Educational attainment reveals that a higher percentage of females (35.18%) than males (29.86%) have some college education or associate degrees. In marital status, a majority of males are married (51.31%) compared to females (48.01%), with higher proportions of widowed (1.88%) and divorced (11.62%) statuses among females. Among male subjects, the prevalence rates were as follows: Chlamydia infection at 41 (0.70%), genital herpes at 126 (2.14%), genital warts at 159 (2.70%), and gonorrhea at 26 (0.44%). Among female subjects, the prevalence rates were: Chlamydia infection at 92 (1.49%), genital herpes at 341 (5.53%), genital warts at 305 (4.95%), gonorrhea at 20 (0.32%), and HPV infection at 556 (9.02%). All feature codes and comments involved in subsequent analysis are shown in Table S1 of Supplemental Files.

**Table 1:** Demographics of datasets

	Male (*n* = 5,890)	Female (*n* = 6,163)
Age (years)	39.04 ± 11.37	39.18 ± 11.36
Education (years)		
Less than 9th grade	338(5.73%)	321(5.21%)
9-11th grade(Include 12th grade with no diploma)	856(14.53%)	734(11.91%)
High school graduate/GED or equivalent	1426(24.21%)	1234(20.02%)
Some college or AA degree	1759(29.86%)	2168(35.18%)
College graduate or above	1511(25.65%)	1706(27.68%)
Marital status		
Married	3022(51.31%)	2959(48.01%)
Widowed	41(0.70%)	116(1.88%)
Divorced	483(8.20%)	716(11.62%)
Separated	164(2.78%)	262(4.25%)
Never married	1483(25.18%)	1447(23.48%)
Living with partner	697(11.83%)	663(10.76%)
Chlamydia	41(0.70%)	92(1.49%)
Genital herpes	126(2.14%)	341(5.53%)
Genital warts	159(2.70%)	305(4.95%)
Gonorrhea	26(0.44%)	20(0.32%)
HPV	/	556(9.02%)

### Classification performance

The CatBoost classifier was trained and validated using ten-fold cross-validation to estimate out-of-sample performance. Evaluation metrics included AUC, recall, accuracy, F1-score, kappa value, and precision. Tables 2 and 3 display the performance of the CatBoost classifier in predicting STI infection risk among male and female populations, respectively. We also compared the performance of 15 models in Table S2-S12 of Supplemental Files. For males, the CatBoost classifier achieved AUC values of 0.9995, 0.9948, 0.9923, and 0.9996 for predicting chlamydia, genital herpes, genital warts, and gonorrhea infections; it also achieved an AUC value of 0.9769 for overall STIs. For females, the classifier attained AUC values of 0.9971, 0.972, 0.9765, 1 for chlamydia, genital herpes, genital warts, and gonorrhea infections; it also reached AUC values of 0.9485 for HPV infection and 0.8819 for overall STIs. The ROC plots and confusion matrix for CatBoost classifier are shown in Figure S1-S4 of Supplemental Files.

**Table 2:** Classification Performance of CatBoost classifier in male populations

Male-Label	Accuracy	AUC	Recall	Prec.	F1	Kappa	MCC
Chlamydia	0.9904	0.9995	0.9978	0.9831	0.9904	0.9807	0.9809
Genital herpes	0.9665	0.9948	0.9841	0.9513	0.9674	0.9329	0.9335
Genital warts	0.9621	0.9923	0.9802	0.9465	0.963	0.9242	0.9249
Gonorrhea	0.9926	0.9996	0.9981	0.9873	0.9927	0.9852	0.9852
STIs	0.923	0.9769	0.9426	0.9061	0.9239	0.8461	0.847

**Table 3:** Classification Performance of CatBoost classifier in female populations

Female-Label	Accuracy	AUC	Recall	Prec.	F1	Kappa	MCC
Chlamydia	0.9792	0.9971	0.9921	0.9675	0.9796	0.9585	0.9588
Genital herpes	0.9137	0.972	0.9322	0.8994	0.9155	0.8275	0.8281
Genital warts	0.9188	0.9765	0.9377	0.9037	0.9202	0.8375	0.8384
Gonorrhea	0.9946	1	0.9988	0.9905	0.9947	0.9893	0.9893
HPV	0.881	0.9485	0.9027	0.8679	0.8848	0.7619	0.7628
STIs	0.7932	0.8819	0.792	0.7904	0.7911	0.5863	0.5865

### Model interpretation: Shapley Additive exPlanations (SHAP)

**Fig. 1:**
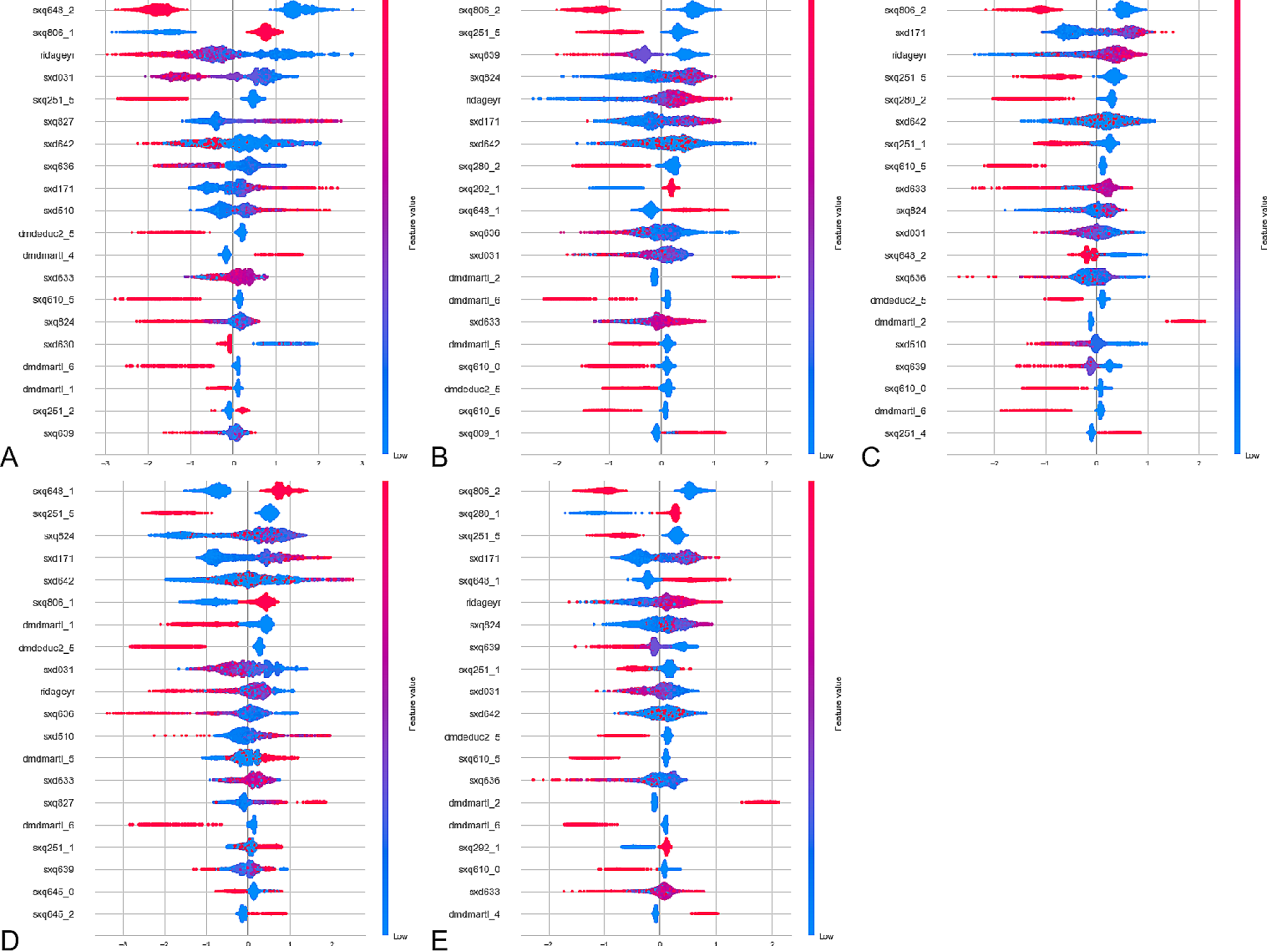
The CatBoost classifiers for predicting chlamydia(**A**), genital herpes(**B**), genital warts(**C**), gonorrhea(**D**), and overall STIs(**E**) based on the SHAP algorithm in male populations

**Fig. 2:**
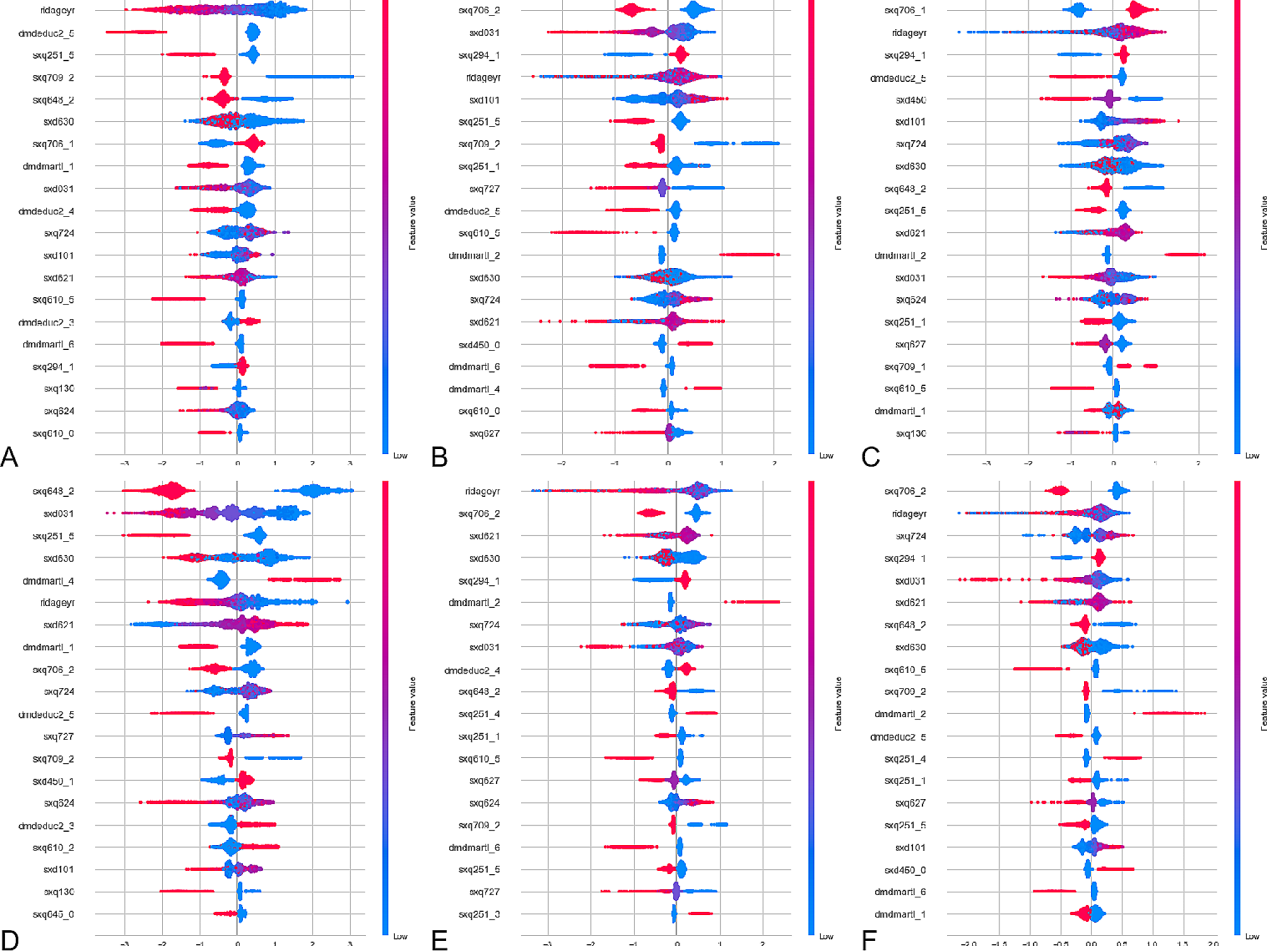
The CatBoost classifiers for predicting chlamydia(**A**), genital herpes(**B**), genital warts(**C**), gonorrhea(**D**), HPV(**E**) and overall STIs(**F**) based on the SHAP algorithm in female populations

Utilizing the SHAP algorithm, the feature ranking interpretation of the CatBoost classifier reveals the top 20 most influential characteristics for predicting outcomes in both male and female populations (Figs. 1 and 2).

In general, the top three significant predictors of male chlamydia infection risk are identified as sxq648_2 (had sex with new partner/year), sxq806_1 (ever had anal sex with a woman), and ridageyr (age in years at screening). The top three important predictors for male genital herpes risk include sxq806_2 (ever had anal sex with a woman), sxq251_5 (times had sex without condom/year), and sxq639 (female performed oral sex/year). For male genital warts risk, the top three important predictors are sxq806_2 (ever had anal sex with a woman), sxd171 (female sex partners/lifetime), and ridageyr (age in years at screening). The top three important predictors for male gonorrhea risk consist of sxq648_1 (had sex with new partner/year), sxq251_5 (times had sex without condom/year), and sxq824 (female vaginal sex partners/life). Lastly, the top three important predictors for total male STI risk include sxq806_2 (ever had anal sex with a woman), sxq280_1 (circumcised or uncircumcised), and sxq251_5 (times had sex without condom/year).

The top three significant predictors of female chlamydia infection risk are identified as ridageyr (age in years at screening), dmdeduc2_5 (education level), and sxq251_5 (times had sex without condom/year). The top three important predictors for female genital herpes risk include sxq706_2 (ever had anal sex with a man), sxd031 (how old when first had sex), and sxq294_1 (sexual identity/attraction). For female genital warts risk, the top three important predictors are sxq706_1 (ever had anal sex with a man), ridageyr (age in years at screening), and sxq294_1 (sexual identity/attraction). The top three important predictors for female gonorrhea risk consist of sxq648_2 (had sex with new partner/year), sxd031 (how old when first had sex), and sxq251_5 (times had sex without condom/year). The top three significant predictors of female HPV infection risk include ridageyr (age in years at screening), sxq706_2 (ever had anal sex with a man) and sxd621(how old when first had oral sex). Lastly, the top three important predictors for total female STI risk include sxq706_2 (ever had anal sex with a man), ridageyr (age in years at screening) and sxq724(male vaginal sex partners/lifetime).

## Discussion

We developed risk prediction models for chlamydia, genital herpes, genital warts, and gonorrhea in male populations, as well as for chlamydia, genital herpes, genital warts, gonorrhea, and HPV infection in female populations using the CatBoost algorithm. The AUC values of these models range from 0.88 to 1, with overall STI prediction AUC values of 0.9769 and 0.8819 for males and females respectively. Lastly, we conducted an interpretability analysis on the models and obtained feature importance rankings for various prediction models.

CatBoost is advantageous for its efficient processing of categorical data and robustness in complex datasets, but these benefits may not be as pronounced in smaller datasets [14]. In contrast, other algorithms such as Random Forest and Light Gradient Boosting Machine show high efficiency in large datasets [15], while Quadratic Discriminant Analysis and Linear Discriminant Analysis perform well with simpler data distributions [16]. Therefore, while CatBoost is a powerful tool, its potential might not have been fully realized in our study. Future research should consider selecting algorithms that better align with the specific characteristics of the dataset to ensure accuracy, efficiency, and interpretability of the model.

In summary, while CatBoost presents a powerful tool for certain types of data, its application in our study might not have leveraged its full potential due to the dataset’s size and nature. Future research could benefit from a more tailored approach in selecting algorithms, where the characteristics of the dataset are closely aligned with the algorithm’s strengths. This approach would ensure not just the accuracy of the model but also the efficiency and interpretability of the results.

Previous studies have employed machine lea rning to predict the risk of STI occurrence. For example, risk prediction tools have been developed to forecast HIV and STIs over the next 12 months [17], demonstrating acceptable performance for HIV (AUC = 0.72), syphilis (AUC = 0.75), gonorrhea (AUC = 0.73), and chlamydia (AUC = 0.67) infection prediction in test datasets. Xianglong Xu et al. [18] developed a machine learning-based STI risk prediction tool, MySTIRisk, which exhibits promising performance on the testing dataset (AUC for HIV = 0.78; AUC for syphilis = 0.84; AUC for gonorrhea = 0.78; AUC for chlamydia = 0.70). Furthermore, it demonstrated stable performance on both external validation data from 2019 (AUC for HIV = 0.79; AUC for syphilis = 0.85; AUC for gonorrhea = 0.81; AUC for chlamydia = 0.69) and data from 2020 to 2021 (AUC for HIV = 0.71; AUC for syphilis = 0.84; AUC for gonorrhea = 0.79; AUC for chlamydia = 0.69). These studies enable individuals to comfortably predict their own risk of HIV and STIs from home. Given that HIV poses higher risks than other STIs, more research has focused on early detection and identification of HIV [7, 19, 20].

Our models show better performance in terms of prediction. We conducted a subgroup analysis based on gender since the likelihood of contracting STIs differs between males and females due to differences in reproductive system structures, aiming to improve our predictive model’s accuracy. Additionally, we carried out an interpretability analysis on our models to assist clinical practitioners in better understanding the models and asking more targeted questions (focusing on the top-ranking features) during actual consultations and screening processes.

Nonetheless, our study presents several limitations: (1) While the ADASYN algorithm was employed for data balancing, which improved performance, it may introduce its own limitations. Specifically, ADASYN can potentially overgeneralize the minority class by creating synthetic samples that do not accurately represent the underlying distribution. This might lead to a model that is less effective in distinguishing between classes in real-world scenarios; (2) Factors influencing STIs may vary across different races. Furthermore, this study did not conduct external validation of the model on distinct datasets; hence, the model’s generalizability has not been tested; (3) The questionnaire data in the database lacks information on HIV and syphilis infection, rendering it impossible to predict associated risks.

To mitigate the aforementioned limitations, future research can implement the following improvements: (1) Explore and apply advanced data balancing techniques that go beyond ADASYN, such as more sophisticated versions of SMOTE algorithms [21] and Generative Adversarial Networks (GANs) [22, 23]. These methods should be carefully evaluated to ensure they do not overgeneralize the minority class and accurately represent the underlying distribution, thereby improving the model’s real-world applicability and robustness; (2) Collect more data from diverse races and regions for external validation and generalization testing of the model; (3) In designing sexual behavior questionnaires, incorporate more data collection on various sexually transmitted diseases to enhance the model’s overall predictive capacity for related infection risks.

In future research, the focus could be directed towards the prevention of STIs in high-risk populations and the intelligent management of STIs-affected individuals. On one hand, developing high-performance early screening models for STIs can expedite the identification of affected populations. On the other hand, for existing diagnosed STIs populations, personalized treatment methods employing artificial intelligence can be adopted to reduce management costs and enhance treatment success rates across different population groups.

## Conclusion

This study found that the CatBoost classifier achieved good classification performance in predicting the risk of different STIs among both male and female populations. The SHAP algorithm identified several important predictors for each STI, with certain demographic characteristics and sexual behaviors being consistently significant across different infections. These findings can inform targeted prevention and intervention efforts to reduce the burden of STIs in the population.

### Electronic supplementary material

Below is the link to the electronic supplementary material.

**Supplementary Material 1: Table S1** All feature codes and comments in datasets. **Table S2** Classification Performance of 15 models for predicting chlamydia in male populations. **Table S3** Classification Performance of 15 models for predicting genital herpes in male populations. **Table S4** Classification Performance of 15 models for predicting genital warts in male populations. **Table S5** Classification Performance of 15 models for predicting gonorrhea in male populations. **Table S6** Classification Performance of 15 models for predicting STIs in male populations. **Table S7** Classification Performance of 15 models for predicting chlamydia in female populations. **Table S8** Classification Performance of 15 models for predicting genital herpes in female populations. **Table S9** Classification Performance of 15 models for predicting genital warts in female populations. **Table S10** Classification Performance of 15 models for predicting gonorrhea in female populations. **Table S11** Classification Performance of 15 models for predicting HPV in female populations. **Table S12** Classification Performance of 15 models for predicting STIs in female populations. **Figure S1**. The CatBoost classifiers for predicting chlamydia(A), genital herpes(B), genital warts(C), gonorrhea(D), and overall STIs(E) based on the confusion matrix in male populations. **Figure S2**. The CatBoost classifiers for predicting chlamydia(A), genital herpes(B), genital warts(C), gonorrhea(D), HPV(E) and overall STIs(F) based on the confusion matrix in female populations. **Figure S3**. The CatBoost classifiers for predicting chlamydia(A), genital herpes(B), genital warts(C), gonorrhea(D), and overall STIs(E) based on the ROC plots in male populations. **Figure S4**. The CatBoost classifiers for predicting chlamydia(A), genital herpes(B), genital warts(C), gonorrhea(D), HPV(E) and overall STIs(F) based on the ROC plots in female populations

## Data Availability

The datasets generated and/or analyzed during the current study are available in the NHANES database (https://www.cdc.gov/nchs/nhanes/index.htm). The questionnaire is located on the NHANES website at: https://wwwn.cdc.gov/nchs/data/nhanes/2009-2010/questionnaires/ai_sxq_f.pdf; https://wwwn.cdc.gov/nchs/data/nhanes/2011-2012/questionnaires/sxq_acasi.pdf; https://wwwn.cdc.gov/nchs/data/nhanes/2013-2014/questionnaires/sxq_acasi_h.pdf; https://wwwn.cdc.gov/nchs/data/nhanes/2015-2016/questionnaires/sxq_acasi_i.pdf. The data used in this study were accessed through a public access repository and no identifiable information was obtained.
