# Supplementary material for: Building gender-specific sexually transmitted infection risk prediction models using CatBoost algorithm and NHANES data
Source: BMC Med Inform Decis Mak. 2024 Jan 24;24:24. doi: 10.1186/s12911-024-02426-1 (PMC10809625; doi:10.1186/s12911-024-02426-1)
Supplement: Supplementary file 1 — Supplementary Material 1: Table S1 All feature codes and comments in datasets. Table S2 Classification Performance of 15 models for predicting chlamydia in male populations. Table S3 Classification Performance of 15 models for predicting genital herpes in male populations. Table S4 Classification Performance of 15 models for predicting genital warts in male populations. Table S5 Classification Performance of 15 models for predicting gonorrhea in male populations. Table S6 Classification Performance of 15 models for predicting STIs in male populations. Table S7 Classification Performance of 15 models for predicting chlamydia in female populations. Table S8 Classification Performance of 15 models for predicting genital herpes in female populations. Table S9 Classification Performance of 15 models for predicting genital warts in female populations. Table S10 Classification Performance of 15 models for predicting gonorrhea in female populations. Table S11 Classification Performance of 15 models for predicting HPV in female populations. Table S12 Classification Performance of 15 models for predicting STIs in female populations. Figure S1. The CatBoost classifiers for predicting chlamydia(A), genital herpes(B), genital warts(C), gonorrhea(D), and overall STIs(E) based on the confusion matrix in male populations. Figure S2. The CatBoost classifiers for predicting chlamydia(A), genital herpes(B), genital warts(C), gonorrhea(D), HPV(E) and overall STIs(F) based on the confusion matrix in female populations. Figure S3. The CatBoost classifiers for predicting chlamydia(A), genital herpes(B), genital warts(C), gonorrhea(D), and overall STIs(E) based on the ROC plots in male populations. Figure S4. The CatBoost classifiers for predicting chlamydia(A), genital herpes(B), genital warts(C), gonorrhea(D), HPV(E) and overall STIs(F) based on the ROC plots in female populations [file 12911_2024_2426_MOESM1_ESM.docx]

**Table S1** All feature codes and comments in datasets

| **Feature Codes** | **Comments** |
| --- | --- |
| riagendr | Gender |
| ridageyr | Age in years at screening |
| dmdeuc2 | Education level |
| dmdmartl | Marital status |
| SXD021 | Ever had vaginal, anal, or oral sex |
| SXQ800 | Ever had vaginal sex with a woman |
| SXQ803 | Ever performed oral sex on a woman |
| SXQ806 | Ever had anal sex with a woman |
| SXQ809 | Ever had any sex with a man: anal, oral |
| SXQ700 | Ever had vaginal sex with a man |
| SXQ703 | Ever performed oral sex on a man |
| SXQ706 | Ever had anal sex with a man |
| SXQ709 | Ever had any kind of sex with a woman |
| SXD031 | How old when first had sex |
| SXD171 | female sex partners/lifetime |
| SXD510 | female sex partners/year |
| SXQ824 | female vaginal sex partners/lifetime |
| SXQ827 | female vaginal sex partners/year |
| SXD633 | Age first performed oral sex on a woman |
| SXQ636 | female performed oral sex/lifetime |
| SXQ639 | female performed oral sex/year |
| SXD642 | Performed oral sex new female (days) |
| SXQ410 | men anal/oral sex partners/lifetime |
| SXQ550 | men anal/oral sex partners/year |
| SXQ836 | men anal sex partners/lifetime |
| SXQ841 | men anal sex partners/year |
| SXQ853 | Ever performed oral sex on a man |
| SXD621 | How old when first had oral sex |
| SXQ624 | male oral sex partners/lifetime |
| SXQ627 | male oral sex partners/year |
| SXD630 | Last performed oral sex new male (days) |
| SXQ645 | Use protection when performing oral sex |
| SXQ648 | Had sex with new partner/year |
| SXQ610 | times had vaginal or anal sex/year |
| SXQ251 | times had sex without condom/year |
| SXQ590 | sex partners five years older/year |
| SXQ600 | sex partners five years younger/year |
| SXD101 | male sex partners/lifetime |
| SXD450 | male sex partners/year |
| SXQ724 | male vaginal sex partners/lifetime |
| SXQ727 | male vaginal sex partners/year |
| SXQ130 | female sex partners/lifetime |
| SXQ490 | female sex partners/year |
| SXQ741 | Ever performed oral sex on a woman |
| SXQ753 | Ever told by doctor, you had HPV |
| SXQ260 | Doctor ever told you had genital herpes |
| SXQ265 | Doctor ever told you had genital warts |
| SXQ267 | Age when told you had genital warts |
| SXQ270 | Doctor ever told you had gonorrhea |
| SXQ272 | Doctor ever told you had chlamydia |
| SXQ280 | Are you circumcised or uncircumcised |
| SXQ292 | Describe sexual orientation (male) |
| SXQ294 | Describe sexual orientation (female) |
| SXQ295 | Describe sexual identity (F) |
| SXQ296 | Describe sexual identity (M) |

**Table S2** Classification Performance of 15 models for predicting chlamydia in male populations

|  | Model | Accuracy | AUC | Recall | Prec. | F1 | Kappa | MCC | TT (Sec) |
| --- | --- | --- | --- | --- | --- | --- | --- | --- | --- |
| rf | Random Forest Classifier | 0.9928 | 0.9998 | 0.9966 | 0.989 | 0.9928 | 0.9856 | 0.9856 | 0.081 |
| xgboost | Extreme Gradient Boosting | 0.9918 | 0.9994 | 0.9975 | 0.9862 | 0.9918 | 0.9837 | 0.9837 | 0.276 |
| et | Extra Trees Classifier | 0.9912 | 0.9996 | 0.9961 | 0.9864 | 0.9912 | 0.9824 | 0.9825 | 0.079 |
| lightgbm | Light Gradient Boosting Machine | 0.9912 | 0.9996 | 0.9968 | 0.9857 | 0.9912 | 0.9824 | 0.9825 | 0.035 |
| catboost | CatBoost Classifier | 0.9904 | 0.9995 | 0.9978 | 0.9831 | 0.9904 | 0.9807 | 0.9809 | 0.733 |
| dt | Decision Tree Classifier | 0.9794 | 0.9795 | 0.9899 | 0.9693 | 0.9795 | 0.9588 | 0.959 | 0.02 |
| gbc | Gradient Boosting Classifier | 0.9745 | 0.9948 | 0.9939 | 0.9566 | 0.9748 | 0.949 | 0.9498 | 0.179 |
| ada | Ada Boost Classifier | 0.9596 | 0.9872 | 0.974 | 0.9463 | 0.9599 | 0.9193 | 0.9197 | 0.061 |
| knn | K Neighbors Classifier | 0.9474 | 0.9796 | 0.9717 | 0.9263 | 0.9484 | 0.8949 | 0.8962 | 0.232 |
| ridge | Ridge Classifier | 0.92 | 0 | 0.9553 | 0.8915 | 0.9222 | 0.84 | 0.8423 | 0.017 |
| lda | Linear Discriminant Analysis | 0.9191 | 0.9752 | 0.955 | 0.8902 | 0.9215 | 0.8383 | 0.8406 | 0.047 |
| lr | Logistic Regression | 0.8339 | 0.8913 | 0.8556 | 0.8184 | 0.8364 | 0.6678 | 0.6688 | 0.487 |
| nb | Naive Bayes | 0.6664 | 0.8661 | 0.9332 | 0.6069 | 0.7354 | 0.3352 | 0.3961 | 0.017 |
| qda | Quadratic Discriminant Analysis | 0.6021 | 0.6 | 0.2906 | 0.7826 | 0.402 | 0.2007 | 0.2588 | 0.029 |
| svm | SVM - Linear Kernel | 0.5894 | 0 | 0.7438 | 0.455 | 0.5638 | 0.1802 | 0.2198 | 0.071 |
| dummy | Dummy Classifier | 0.5034 | 0.5 | 0 | 0 | 0 | 0 | 0 | 0.013 |

**Table S3** Classification Performance of 15 models for predicting genital herpes in male populations

|  | Model | Accuracy | AUC | Recall | Prec. | F1 | Kappa | MCC | TT (Sec) |
| --- | --- | --- | --- | --- | --- | --- | --- | --- | --- |
| rf | Random Forest Classifier | 0.9743 | 0.9969 | 0.9846 | 0.9653 | 0.9748 | 0.9485 | 0.9488 | 0.09 |
| xgboost | Extreme Gradient Boosting | 0.9712 | 0.9965 | 0.9846 | 0.9595 | 0.9718 | 0.9423 | 0.9427 | 0.407 |
| catboost | CatBoost Classifier | 0.9665 | 0.9948 | 0.9841 | 0.9513 | 0.9674 | 0.9329 | 0.9335 | 0.934 |
| et | Extra Trees Classifier | 0.9657 | 0.9952 | 0.977 | 0.9563 | 0.9664 | 0.9314 | 0.9318 | 0.191 |
| lightgbm | Light Gradient Boosting Machine | 0.9648 | 0.9948 | 0.9814 | 0.9508 | 0.9658 | 0.9297 | 0.9303 | 0.059 |
| dt | Decision Tree Classifier | 0.935 | 0.9347 | 0.9616 | 0.9144 | 0.9373 | 0.8699 | 0.8713 | 0.02 |
| gbc | Gradient Boosting Classifier | 0.9017 | 0.9643 | 0.9314 | 0.8812 | 0.9055 | 0.8033 | 0.8049 | 0.219 |
| knn | K Neighbors Classifier | 0.8976 | 0.9594 | 0.9672 | 0.8508 | 0.9052 | 0.7949 | 0.8027 | 0.254 |
| ada | Ada Boost Classifier | 0.8659 | 0.9346 | 0.883 | 0.8565 | 0.8694 | 0.7318 | 0.7323 | 0.061 |
| ridge | Ridge Classifier | 0.8355 | 0 | 0.8737 | 0.8146 | 0.8429 | 0.6707 | 0.6728 | 0.018 |
| lda | Linear Discriminant Analysis | 0.8351 | 0.9103 | 0.8729 | 0.8145 | 0.8425 | 0.67 | 0.672 | 0.059 |
| lr | Logistic Regression | 0.6647 | 0.7063 | 0.7356 | 0.6491 | 0.6888 | 0.3283 | 0.3325 | 0.467 |
| qda | Quadratic Discriminant Analysis | 0.58 | 0.5814 | 0.4567 | 0.6229 | 0.5148 | 0.1623 | 0.1744 | 0.031 |
| nb | Naive Bayes | 0.5735 | 0.7406 | 0.2431 | 0.732 | 0.3613 | 0.1531 | 0.2045 | 0.017 |
| svm | SVM - Linear Kernel | 0.5106 | 0 | 0.3998 | 0.6453 | 0.3114 | 0.0234 | 0.0327 | 0.079 |
| dummy | Dummy Classifier | 0.5055 | 0.5 | 1 | 0.5055 | 0.6715 | 0 | 0 | 0.013 |

**Table S4** Classification Performance of 15 models for predicting genital warts in male populations

|  | Model | Accuracy | AUC | Recall | Prec. | F1 | Kappa | MCC | TT (Sec) |
| --- | --- | --- | --- | --- | --- | --- | --- | --- | --- |
| rf | Random Forest Classifier | 0.969 | 0.9954 | 0.9799 | 0.9593 | 0.9694 | 0.9379 | 0.9382 | 0.09 |
| xgboost | Extreme Gradient Boosting | 0.9672 | 0.9946 | 0.9802 | 0.9559 | 0.9678 | 0.9344 | 0.9349 | 0.244 |
| et | Extra Trees Classifier | 0.963 | 0.9942 | 0.9757 | 0.952 | 0.9636 | 0.926 | 0.9264 | 0.087 |
| catboost | CatBoost Classifier | 0.9621 | 0.9923 | 0.9802 | 0.9465 | 0.963 | 0.9242 | 0.9249 | 0.736 |
| lightgbm | Light Gradient Boosting Machine | 0.9611 | 0.9914 | 0.9774 | 0.947 | 0.9619 | 0.9222 | 0.9228 | 0.037 |
| dt | Decision Tree Classifier | 0.9222 | 0.9221 | 0.9536 | 0.8982 | 0.925 | 0.8444 | 0.8462 | 0.021 |
| gbc | Gradient Boosting Classifier | 0.89 | 0.9557 | 0.9122 | 0.8744 | 0.8928 | 0.7799 | 0.7807 | 0.184 |
| knn | K Neighbors Classifier | 0.8887 | 0.9526 | 0.9727 | 0.8337 | 0.8978 | 0.7772 | 0.7885 | 0.244 |
| ada | Ada Boost Classifier | 0.8482 | 0.9269 | 0.8713 | 0.8343 | 0.8522 | 0.6963 | 0.6973 | 0.058 |
| lda | Linear Discriminant Analysis | 0.8384 | 0.9188 | 0.8762 | 0.8161 | 0.8449 | 0.6766 | 0.6788 | 0.04 |
| ridge | Ridge Classifier | 0.8375 | 0 | 0.8757 | 0.8151 | 0.8442 | 0.6748 | 0.6771 | 0.017 |
| nb | Naive Bayes | 0.6681 | 0.7529 | 0.7897 | 0.6466 | 0.7045 | 0.3354 | 0.3524 | 0.016 |
| lr | Logistic Regression | 0.6605 | 0.6751 | 0.7636 | 0.6366 | 0.6933 | 0.3203 | 0.3284 | 0.431 |
| qda | Quadratic Discriminant Analysis | 0.5654 | 0.5658 | 0.5139 | 0.6039 | 0.5265 | 0.1314 | 0.1463 | 0.027 |
| svm | SVM - Linear Kernel | 0.5414 | 0 | 0.3541 | 0.62 | 0.2949 | 0.085 | 0.1146 | 0.073 |
| dummy | Dummy Classifier | 0.5025 | 0.5 | 1 | 0.5025 | 0.6689 | 0 | 0 | 0.013 |

**Table S5** Classification Performance of 15 models for predicting gonorrhea in male populations

|  | Model | Accuracy | AUC | Recall | Prec. | F1 | Kappa | MCC | TT (Sec) |
| --- | --- | --- | --- | --- | --- | --- | --- | --- | --- |
| rf | Random Forest Classifier | 0.9959 | 0.9997 | 0.9976 | 0.9942 | 0.9959 | 0.9917 | 0.9917 | 0.083 |
| et | Extra Trees Classifier | 0.9956 | 0.9994 | 0.9966 | 0.9947 | 0.9957 | 0.9912 | 0.9912 | 0.081 |
| lightgbm | Light Gradient Boosting Machine | 0.9944 | 0.9997 | 0.9976 | 0.9914 | 0.9945 | 0.9888 | 0.9888 | 0.037 |
| xgboost | Extreme Gradient Boosting | 0.9937 | 0.9998 | 0.9973 | 0.9902 | 0.9937 | 0.9873 | 0.9874 | 0.285 |
| catboost | CatBoost Classifier | 0.9926 | 0.9996 | 0.9981 | 0.9873 | 0.9927 | 0.9852 | 0.9852 | 0.757 |
| dt | Decision Tree Classifier | 0.9834 | 0.9834 | 0.9932 | 0.9744 | 0.9837 | 0.9669 | 0.9671 | 0.021 |
| gbc | Gradient Boosting Classifier | 0.9707 | 0.995 | 0.9947 | 0.9495 | 0.9716 | 0.9413 | 0.9424 | 0.19 |
| knn | K Neighbors Classifier | 0.9395 | 0.9735 | 0.9722 | 0.9133 | 0.9418 | 0.879 | 0.881 | 0.252 |
| ada | Ada Boost Classifier | 0.9325 | 0.9756 | 0.9579 | 0.9124 | 0.9345 | 0.8649 | 0.8661 | 0.064 |
| ridge | Ridge Classifier | 0.8865 | 0 | 0.9221 | 0.862 | 0.891 | 0.7728 | 0.7748 | 0.017 |
| lda | Linear Discriminant Analysis | 0.8861 | 0.9535 | 0.9224 | 0.8613 | 0.8907 | 0.7721 | 0.7741 | 0.05 |
| nb | Naive Bayes | 0.7555 | 0.83 | 0.8605 | 0.7135 | 0.7798 | 0.5103 | 0.5227 | 0.017 |
| lr | Logistic Regression | 0.7353 | 0.8201 | 0.7108 | 0.7503 | 0.7299 | 0.4708 | 0.4715 | 0.451 |
| qda | Quadratic Discriminant Analysis | 0.5815 | 0.5826 | 0.4079 | 0.6338 | 0.4906 | 0.1649 | 0.1781 | 0.031 |
| svm | SVM - Linear Kernel | 0.5159 | 0 | 0.5969 | 0.6625 | 0.4384 | 0.0306 | 0.0635 | 0.1 |
| dummy | Dummy Classifier | 0.5033 | 0.5 | 1 | 0.5033 | 0.6696 | 0 | 0 | 0.013 |

**Table S6** Classification Performance of 15 models for predicting STIs in male populations

|  | Model | Accuracy | AUC | Recall | Prec. | F1 | Kappa | MCC | TT (Sec) |
| --- | --- | --- | --- | --- | --- | --- | --- | --- | --- |
| xgboost | Extreme Gradient Boosting | 0.9398 | 0.982 | 0.9564 | 0.9247 | 0.9403 | 0.8796 | 0.8802 | 0.25 |
| rf | Random Forest Classifier | 0.9385 | 0.9855 | 0.9534 | 0.925 | 0.9389 | 0.8771 | 0.8775 | 0.197 |
| et | Extra Trees Classifier | 0.9277 | 0.9807 | 0.9442 | 0.9132 | 0.9283 | 0.8555 | 0.8561 | 0.176 |
| lightgbm | Light Gradient Boosting Machine | 0.9237 | 0.9758 | 0.938 | 0.9107 | 0.9241 | 0.8473 | 0.8478 | 0.034 |
| catboost | CatBoost Classifier | 0.923 | 0.9769 | 0.9426 | 0.9061 | 0.9239 | 0.8461 | 0.847 | 0.727 |
| dt | Decision Tree Classifier | 0.8784 | 0.8787 | 0.9139 | 0.8519 | 0.8817 | 0.757 | 0.7591 | 0.02 |
| gbc | Gradient Boosting Classifier | 0.8565 | 0.931 | 0.8776 | 0.8402 | 0.8584 | 0.713 | 0.7138 | 0.285 |
| knn | K Neighbors Classifier | 0.8502 | 0.9252 | 0.9536 | 0.7887 | 0.8633 | 0.7009 | 0.7166 | 0.233 |
| ada | Ada Boost Classifier | 0.8262 | 0.9055 | 0.846 | 0.8118 | 0.8284 | 0.6525 | 0.6535 | 0.094 |
| ridge | Ridge Classifier | 0.823 | 0 | 0.8463 | 0.8067 | 0.8259 | 0.6462 | 0.6472 | 0.018 |
| lda | Linear Discriminant Analysis | 0.8227 | 0.8956 | 0.8466 | 0.8059 | 0.8256 | 0.6454 | 0.6465 | 0.031 |
| lr | Logistic Regression | 0.6557 | 0.6923 | 0.7341 | 0.6321 | 0.6791 | 0.3123 | 0.3165 | 0.434 |
| svm | SVM - Linear Kernel | 0.544 | 0 | 0.3575 | 0.6263 | 0.3054 | 0.0844 | 0.1192 | 0.051 |
| nb | Naive Bayes | 0.5381 | 0.7564 | 0.1345 | 0.6663 | 0.2234 | 0.0702 | 0.1149 | 0.016 |
| qda | Quadratic Discriminant Analysis | 0.5379 | 0.5379 | 0.5459 | 0.5366 | 0.5373 | 0.0759 | 0.0777 | 0.031 |
| dummy | Dummy Classifier | 0.5041 | 0.5 | 0 | 0 | 0 | 0 | 0 | 0.013 |

**Table S7** Classification Performance of 15 models for predicting chlamydia in female populations

|  | Model | Accuracy | AUC | Recall | Prec. | F1 | Kappa | MCC | TT (Sec) |
| --- | --- | --- | --- | --- | --- | --- | --- | --- | --- |
| rf | Random Forest Classifier | 0.9863 | 0.9989 | 0.9939 | 0.9791 | 0.9865 | 0.9725 | 0.9727 | 0.089 |
| et | Extra Trees Classifier | 0.9843 | 0.9986 | 0.9909 | 0.9782 | 0.9845 | 0.9685 | 0.9687 | 0.093 |
| xgboost | Extreme Gradient Boosting | 0.9832 | 0.998 | 0.9923 | 0.9749 | 0.9835 | 0.9664 | 0.9666 | 0.29 |
| lightgbm | Light Gradient Boosting Machine | 0.9802 | 0.9973 | 0.9904 | 0.9708 | 0.9805 | 0.9603 | 0.9605 | 0.033 |
| catboost | CatBoost Classifier | 0.9792 | 0.9971 | 0.9921 | 0.9675 | 0.9796 | 0.9585 | 0.9588 | 0.617 |
| dt | Decision Tree Classifier | 0.9581 | 0.958 | 0.9814 | 0.9384 | 0.9594 | 0.9162 | 0.9173 | 0.165 |
| knn | K Neighbors Classifier | 0.9303 | 0.9746 | 0.9793 | 0.8928 | 0.934 | 0.8605 | 0.8647 | 0.081 |
| gbc | Gradient Boosting Classifier | 0.9254 | 0.978 | 0.9441 | 0.9113 | 0.9273 | 0.8507 | 0.8515 | 0.192 |
| ada | Ada Boost Classifier | 0.8822 | 0.9558 | 0.8967 | 0.8728 | 0.8846 | 0.7643 | 0.7647 | 0.064 |
| ridge | Ridge Classifier | 0.882 | 0 | 0.9 | 0.8701 | 0.8848 | 0.7638 | 0.7644 | 0.017 |
| lda | Linear Discriminant Analysis | 0.8818 | 0.9497 | 0.9002 | 0.8698 | 0.8847 | 0.7636 | 0.7642 | 0.059 |
| lr | Logistic Regression | 0.7056 | 0.7629 | 0.7308 | 0.6984 | 0.7141 | 0.411 | 0.4116 | 0.078 |
| nb | Naive Bayes | 0.6632 | 0.7811 | 0.8664 | 0.6182 | 0.7215 | 0.3246 | 0.3553 | 0.154 |
| qda | Quadratic Discriminant Analysis | 0.6262 | 0.6265 | 0.5732 | 0.6523 | 0.6049 | 0.2528 | 0.2584 | 0.03 |
| svm | SVM - Linear Kernel | 0.5039 | 0 | 0.3951 | 0.3342 | 0.2999 | 0.0096 | 0.0126 | 0.274 |
| dummy | Dummy Classifier | 0.5034 | 0.5 | 1 | 0.5034 | 0.6697 | 0 | 0 | 0.014 |

**Table S8** Classification Performance of 15 models for predicting genital herpes in female populations

|  | Model | Accuracy | AUC | Recall | Prec. | F1 | Kappa | MCC | TT (Sec) |
| --- | --- | --- | --- | --- | --- | --- | --- | --- | --- |
| rf | Random Forest Classifier | 0.9367 | 0.9844 | 0.9537 | 0.9228 | 0.9379 | 0.8734 | 0.8741 | 0.101 |
| xgboost | Extreme Gradient Boosting | 0.9277 | 0.9784 | 0.9488 | 0.9107 | 0.9293 | 0.8553 | 0.8562 | 0.25 |
| et | Extra Trees Classifier | 0.9263 | 0.9798 | 0.9405 | 0.915 | 0.9275 | 0.8527 | 0.8531 | 0.106 |
| catboost | CatBoost Classifier | 0.9137 | 0.972 | 0.9322 | 0.8994 | 0.9155 | 0.8275 | 0.8281 | 0.626 |
| lightgbm | Light Gradient Boosting Machine | 0.9081 | 0.9697 | 0.9271 | 0.8937 | 0.91 | 0.8162 | 0.817 | 0.034 |
| dt | Decision Tree Classifier | 0.864 | 0.8639 | 0.9078 | 0.8353 | 0.8699 | 0.728 | 0.731 | 0.02 |
| knn | K Neighbors Classifier | 0.8621 | 0.9386 | 0.9705 | 0.7981 | 0.8758 | 0.724 | 0.7418 | 0.248 |
| gbc | Gradient Boosting Classifier | 0.8348 | 0.9219 | 0.8498 | 0.8258 | 0.8375 | 0.6696 | 0.6701 | 0.168 |
| ridge | Ridge Classifier | 0.811 | 0 | 0.8166 | 0.8084 | 0.8123 | 0.622 | 0.6223 | 0.016 |
| ada | Ada Boost Classifier | 0.8106 | 0.8967 | 0.8232 | 0.8036 | 0.8132 | 0.6212 | 0.6216 | 0.057 |
| lda | Linear Discriminant Analysis | 0.8104 | 0.8948 | 0.8163 | 0.8076 | 0.8118 | 0.6208 | 0.621 | 0.046 |
| lr | Logistic Regression | 0.6441 | 0.7013 | 0.7759 | 0.6186 | 0.6853 | 0.2879 | 0.3022 | 0.445 |
| nb | Naive Bayes | 0.6395 | 0.7275 | 0.8376 | 0.6009 | 0.6995 | 0.2784 | 0.3035 | 0.017 |
| svm | SVM - Linear Kernel | 0.5889 | 0 | 0.5763 | 0.5814 | 0.4968 | 0.1779 | 0.1964 | 0.075 |
| qda | Quadratic Discriminant Analysis | 0.5625 | 0.5627 | 0.4788 | 0.5808 | 0.52 | 0.1253 | 0.1293 | 0.024 |
| dummy | Dummy Classifier | 0.5009 | 0.5 | 1 | 0.5009 | 0.6675 | 0 | 0 | 0.014 |

**Table S9** Classification Performance of 15 models for predicting genital warts in female populations

|  | Model | Accuracy | AUC | Recall | Prec. | F1 | Kappa | MCC | TT (Sec) |
| --- | --- | --- | --- | --- | --- | --- | --- | --- | --- |
| rf | Random Forest Classifier | 0.9406 | 0.9857 | 0.9536 | 0.9295 | 0.9413 | 0.8812 | 0.8816 | 0.094 |
| xgboost | Extreme Gradient Boosting | 0.9338 | 0.9823 | 0.9506 | 0.9195 | 0.9348 | 0.8675 | 0.8681 | 0.219 |
| et | Extra Trees Classifier | 0.9288 | 0.981 | 0.9406 | 0.9189 | 0.9295 | 0.8575 | 0.858 | 0.1 |
| catboost | CatBoost Classifier | 0.9188 | 0.9765 | 0.9377 | 0.9037 | 0.9202 | 0.8375 | 0.8384 | 0.643 |
| lightgbm | Light Gradient Boosting Machine | 0.9134 | 0.9736 | 0.9269 | 0.9024 | 0.9144 | 0.8268 | 0.8272 | 0.033 |
| dt | Decision Tree Classifier | 0.8814 | 0.8815 | 0.9115 | 0.8594 | 0.8847 | 0.7629 | 0.7644 | 0.02 |
| knn | K Neighbors Classifier | 0.8637 | 0.9419 | 0.9624 | 0.8039 | 0.8758 | 0.7276 | 0.7425 | 0.245 |
| gbc | Gradient Boosting Classifier | 0.853 | 0.9297 | 0.8563 | 0.8505 | 0.8532 | 0.706 | 0.7062 | 0.157 |
| ada | Ada Boost Classifier | 0.8238 | 0.9036 | 0.8262 | 0.8221 | 0.8239 | 0.6477 | 0.648 | 0.053 |
| lda | Linear Discriminant Analysis | 0.8037 | 0.8912 | 0.8096 | 0.8 | 0.8046 | 0.6074 | 0.6078 | 0.031 |
| ridge | Ridge Classifier | 0.8032 | 0 | 0.8084 | 0.7999 | 0.8039 | 0.6065 | 0.6068 | 0.017 |
| lr | Logistic Regression | 0.674 | 0.7235 | 0.7744 | 0.6483 | 0.7037 | 0.3483 | 0.3579 | 0.435 |
| nb | Naive Bayes | 0.6279 | 0.755 | 0.8902 | 0.5849 | 0.7051 | 0.2566 | 0.3023 | 0.015 |
| qda | Quadratic Discriminant Analysis | 0.5598 | 0.5597 | 0.4598 | 0.5841 | 0.5006 | 0.1194 | 0.1271 | 0.023 |
| svm | SVM - Linear Kernel | 0.5442 | 0 | 0.5673 | 0.5316 | 0.4579 | 0.0883 | 0.1026 | 0.069 |
| dummy | Dummy Classifier | 0.5009 | 0.5 | 0 | 0 | 0 | 0 | 0 | 0.013 |

**Table S10** Classification Performance of 15 models for predicting gonorrhea in female populations

|  | Model | Accuracy | AUC | Recall | Prec. | F1 | Kappa | MCC | TT (Sec) |
| --- | --- | --- | --- | --- | --- | --- | --- | --- | --- |
| et | Extra Trees Classifier | 0.9969 | 0.9999 | 0.9981 | 0.9956 | 0.9969 | 0.9937 | 0.9937 | 0.078 |
| rf | Random Forest Classifier | 0.9965 | 0.9999 | 0.9979 | 0.9951 | 0.9965 | 0.993 | 0.993 | 0.087 |
| xgboost | Extreme Gradient Boosting | 0.9958 | 0.9999 | 0.9991 | 0.9926 | 0.9958 | 0.9916 | 0.9917 | 0.296 |
| lightgbm | Light Gradient Boosting Machine | 0.9952 | 0.9999 | 0.9988 | 0.9917 | 0.9952 | 0.9905 | 0.9905 | 0.034 |
| catboost | CatBoost Classifier | 0.9946 | 1 | 0.9988 | 0.9905 | 0.9947 | 0.9893 | 0.9893 | 0.638 |
| dt | Decision Tree Classifier | 0.9902 | 0.9902 | 0.9977 | 0.983 | 0.9903 | 0.9805 | 0.9806 | 0.02 |
| gbc | Gradient Boosting Classifier | 0.9819 | 0.9981 | 0.9986 | 0.9662 | 0.9821 | 0.9637 | 0.9643 | 0.202 |
| ada | Ada Boost Classifier | 0.9727 | 0.993 | 0.989 | 0.9576 | 0.973 | 0.9453 | 0.9459 | 0.064 |
| knn | K Neighbors Classifier | 0.9556 | 0.9831 | 0.979 | 0.9351 | 0.9565 | 0.9112 | 0.9122 | 0.24 |
| ridge | Ridge Classifier | 0.9516 | 0 | 0.9916 | 0.918 | 0.9534 | 0.9033 | 0.9062 | 0.017 |
| lda | Linear Discriminant Analysis | 0.9516 | 0.9874 | 0.9916 | 0.918 | 0.9534 | 0.9033 | 0.9062 | 0.055 |
| lr | Logistic Regression | 0.8084 | 0.8871 | 0.822 | 0.7997 | 0.8105 | 0.6169 | 0.6174 | 0.517 |
| nb | Naive Bayes | 0.7397 | 0.8702 | 0.9748 | 0.6625 | 0.7888 | 0.4801 | 0.5439 | 0.016 |
| qda | Quadratic Discriminant Analysis | 0.635 | 0.6343 | 0.3751 | 0.782 | 0.502 | 0.269 | 0.3158 | 0.033 |
| svm | SVM - Linear Kernel | 0.5692 | 0 | 0.5497 | 0.3476 | 0.4234 | 0.1379 | 0.1686 | 0.085 |
| dummy | Dummy Classifier | 0.5014 | 0.5 | 0 | 0 | 0 | 0 | 0 | 0.013 |

**Table S11** Classification Performance of 15 models for predicting HPV in female populations

|  | Model | Accuracy | AUC | Recall | Prec. | F1 | Kappa | MCC | TT (Sec) |
| --- | --- | --- | --- | --- | --- | --- | --- | --- | --- |
| rf | Random Forest Classifier | 0.8979 | 0.9669 | 0.9245 | 0.8799 | 0.9016 | 0.7956 | 0.7968 | 0.101 |
| xgboost | Extreme Gradient Boosting | 0.8959 | 0.9587 | 0.9196 | 0.8802 | 0.8994 | 0.7916 | 0.7925 | 0.243 |
| et | Extra Trees Classifier | 0.8911 | 0.9583 | 0.9099 | 0.8793 | 0.8943 | 0.7821 | 0.7827 | 0.104 |
| catboost | CatBoost Classifier | 0.881 | 0.9485 | 0.9027 | 0.8679 | 0.8848 | 0.7619 | 0.7628 | 0.619 |
| lightgbm | Light Gradient Boosting Machine | 0.8789 | 0.9467 | 0.8994 | 0.8665 | 0.8826 | 0.7577 | 0.7584 | 0.034 |
| dt | Decision Tree Classifier | 0.8326 | 0.832 | 0.8751 | 0.8096 | 0.841 | 0.6647 | 0.6671 | 0.02 |
| knn | K Neighbors Classifier | 0.8204 | 0.9096 | 0.9553 | 0.7549 | 0.8433 | 0.6395 | 0.6642 | 0.251 |
| gbc | Gradient Boosting Classifier | 0.8096 | 0.896 | 0.8235 | 0.805 | 0.814 | 0.619 | 0.6193 | 0.168 |
| ada | Ada Boost Classifier | 0.79 | 0.8753 | 0.8026 | 0.7873 | 0.7946 | 0.5798 | 0.5804 | 0.056 |
| ridge | Ridge Classifier | 0.7846 | 0 | 0.7917 | 0.7851 | 0.7882 | 0.569 | 0.5693 | 0.017 |
| lda | Linear Discriminant Analysis | 0.7844 | 0.8628 | 0.7917 | 0.7849 | 0.7881 | 0.5688 | 0.5691 | 0.039 |
| nb | Naive Bayes | 0.6596 | 0.7212 | 0.7847 | 0.6321 | 0.6997 | 0.3171 | 0.328 | 0.017 |
| lr | Logistic Regression | 0.6451 | 0.6726 | 0.7587 | 0.6238 | 0.6835 | 0.2883 | 0.2972 | 0.418 |
| qda | Quadratic Discriminant Analysis | 0.5453 | 0.5471 | 0.3938 | 0.576 | 0.4648 | 0.0939 | 0.1001 | 0.025 |
| svm | SVM - Linear Kernel | 0.545 | 0 | 0.4514 | 0.6876 | 0.3641 | 0.0927 | 0.127 | 0.08 |
| dummy | Dummy Classifier | 0.506 | 0.5 | 1 | 0.506 | 0.6719 | 0 | 0 | 0.013 |

**Table S12** Classification Performance of 15 models for predicting STIs in female populations

|  | Model | Accuracy | AUC | Recall | Prec. | F1 | Kappa | MCC | TT (Sec) |
| --- | --- | --- | --- | --- | --- | --- | --- | --- | --- |
| rf | Random Forest Classifier | 0.8219 | 0.9075 | 0.8416 | 0.807 | 0.8237 | 0.644 | 0.6449 | 0.089 |
| xgboost | Extreme Gradient Boosting | 0.8118 | 0.8967 | 0.8199 | 0.8039 | 0.8116 | 0.6236 | 0.6241 | 0.219 |
| et | Extra Trees Classifier | 0.8009 | 0.8919 | 0.816 | 0.7891 | 0.8021 | 0.602 | 0.6027 | 0.094 |
| lightgbm | Light Gradient Boosting Machine | 0.7952 | 0.8806 | 0.7917 | 0.7939 | 0.7926 | 0.5903 | 0.5906 | 0.033 |
| catboost | CatBoost Classifier | 0.7932 | 0.8819 | 0.792 | 0.7904 | 0.7911 | 0.5863 | 0.5865 | 0.599 |
| knn | K Neighbors Classifier | 0.7609 | 0.8374 | 0.9011 | 0.7012 | 0.7886 | 0.5233 | 0.5455 | 0.232 |
| gbc | Gradient Boosting Classifier | 0.7606 | 0.8418 | 0.7558 | 0.7591 | 0.7573 | 0.5212 | 0.5214 | 0.147 |
| ada | Ada Boost Classifier | 0.7449 | 0.8257 | 0.7427 | 0.7418 | 0.7422 | 0.4897 | 0.4898 | 0.053 |
| lda | Linear Discriminant Analysis | 0.7416 | 0.823 | 0.7268 | 0.7449 | 0.7356 | 0.483 | 0.4833 | 0.034 |
| ridge | Ridge Classifier | 0.7413 | 0 | 0.7259 | 0.745 | 0.7352 | 0.4825 | 0.4828 | 0.017 |
| dt | Decision Tree Classifier | 0.7353 | 0.7356 | 0.7667 | 0.7175 | 0.7412 | 0.4709 | 0.4722 | 0.019 |
| lr | Logistic Regression | 0.6327 | 0.6838 | 0.7889 | 0.5975 | 0.6798 | 0.2679 | 0.2827 | 0.421 |
| nb | Naive Bayes | 0.5965 | 0.7007 | 0.3467 | 0.6833 | 0.4527 | 0.1885 | 0.2174 | 0.005 |
| qda | Quadratic Discriminant Analysis | 0.5395 | 0.5385 | 0.4504 | 0.5594 | 0.477 | 0.0773 | 0.0859 | 0.025 |
| dummy | Dummy Classifier | 0.5055 | 0.5 | 0 | 0 | 0 | 0 | 0 | 0.013 |
| svm | SVM - Linear Kernel | 0.5041 | 0 | 0.4182 | 0.5315 | 0.3236 | 0.0062 | 0.0136 | 0.089 |


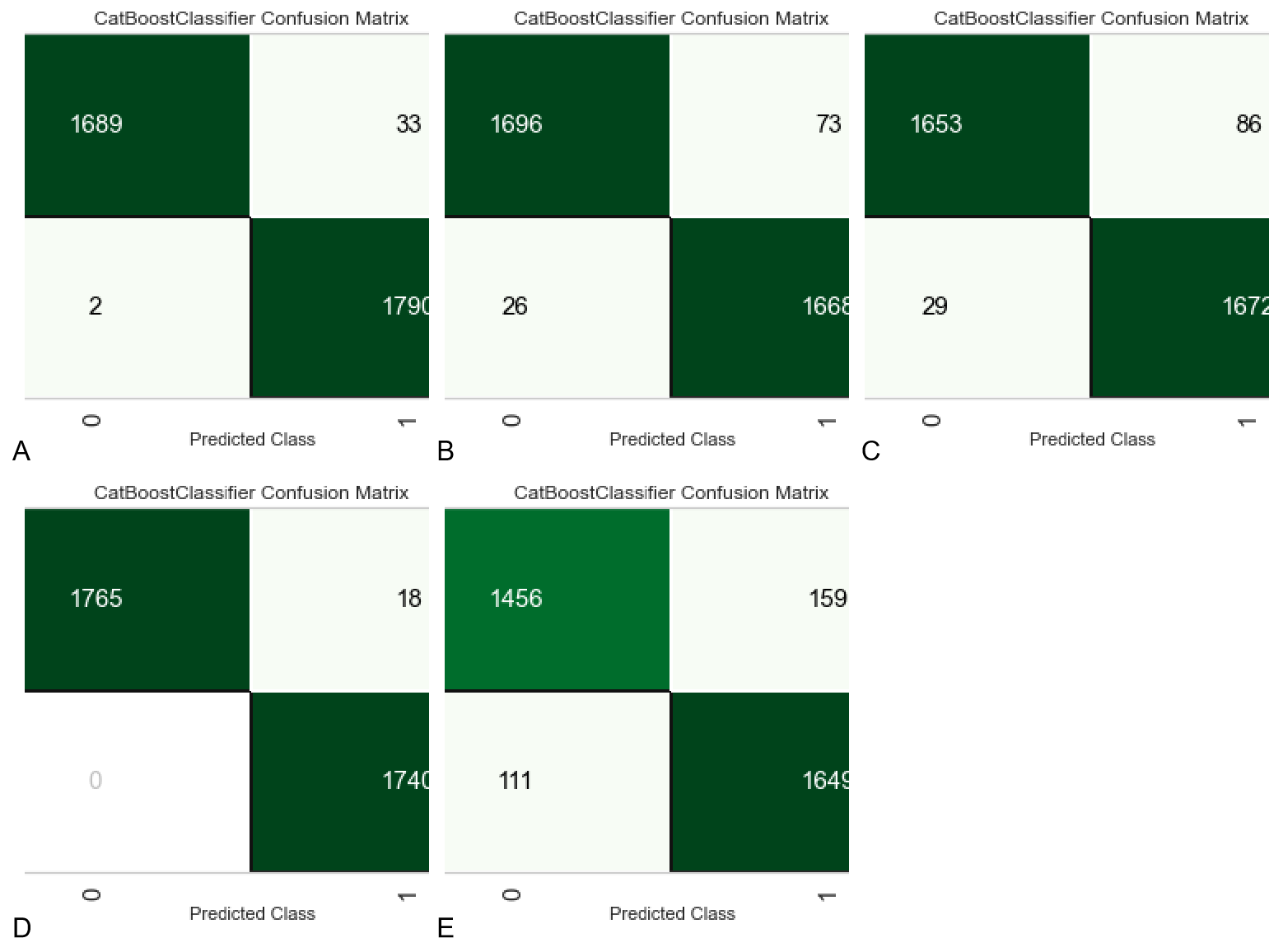


**Figure S1.** The CatBoost classifiers for predicting chlamydia(A), genital herpes(B), genital warts(C), gonorrhea(D), and overall STIs(E) based on the confusion matrix in male populations.


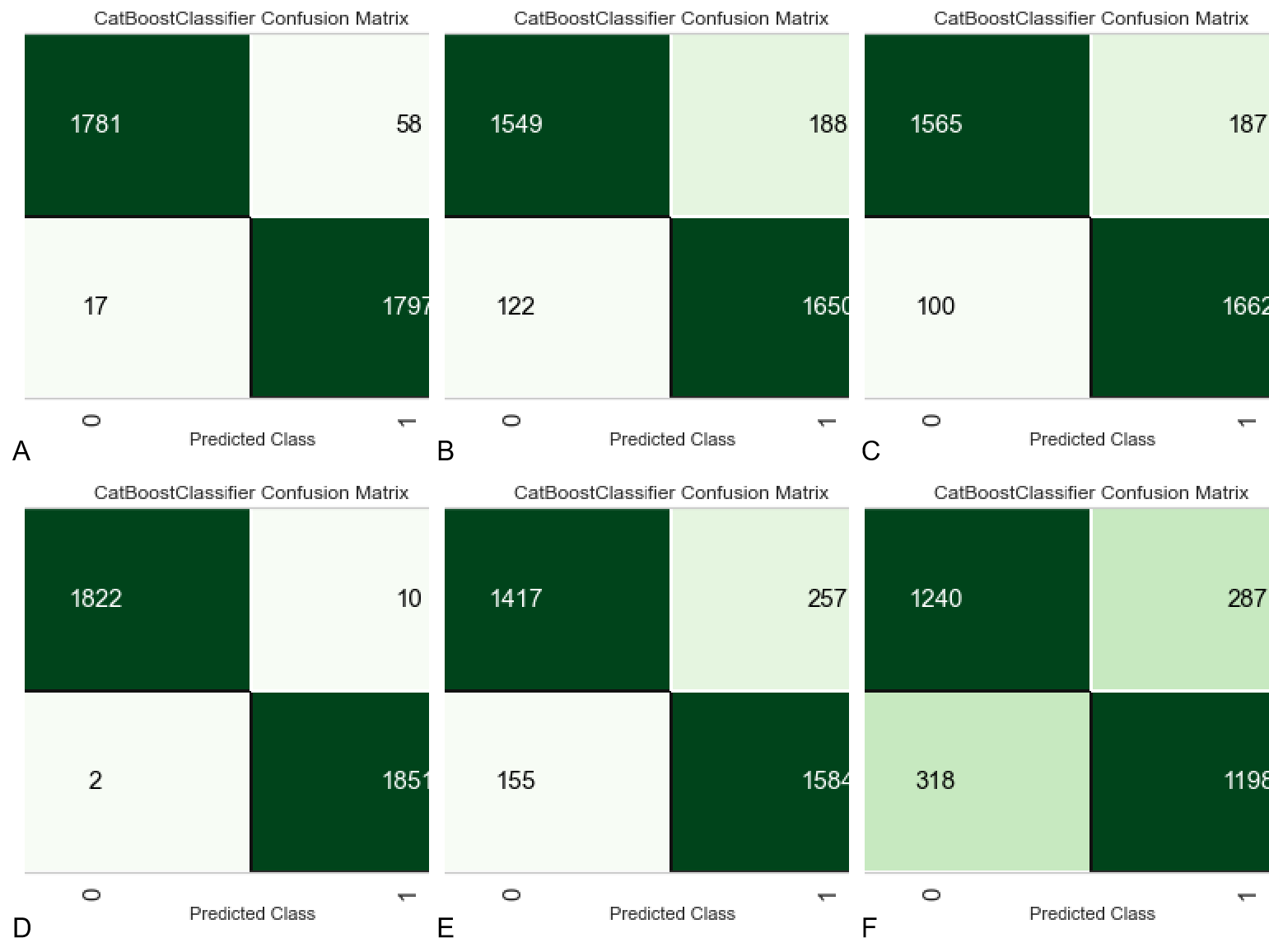


**Figure S2.** The CatBoost classifiers for predicting chlamydia(A), genital herpes(B), genital warts(C), gonorrhea(D), HPV(E) and overall STIs(F) based on the confusion matrix in female populations.


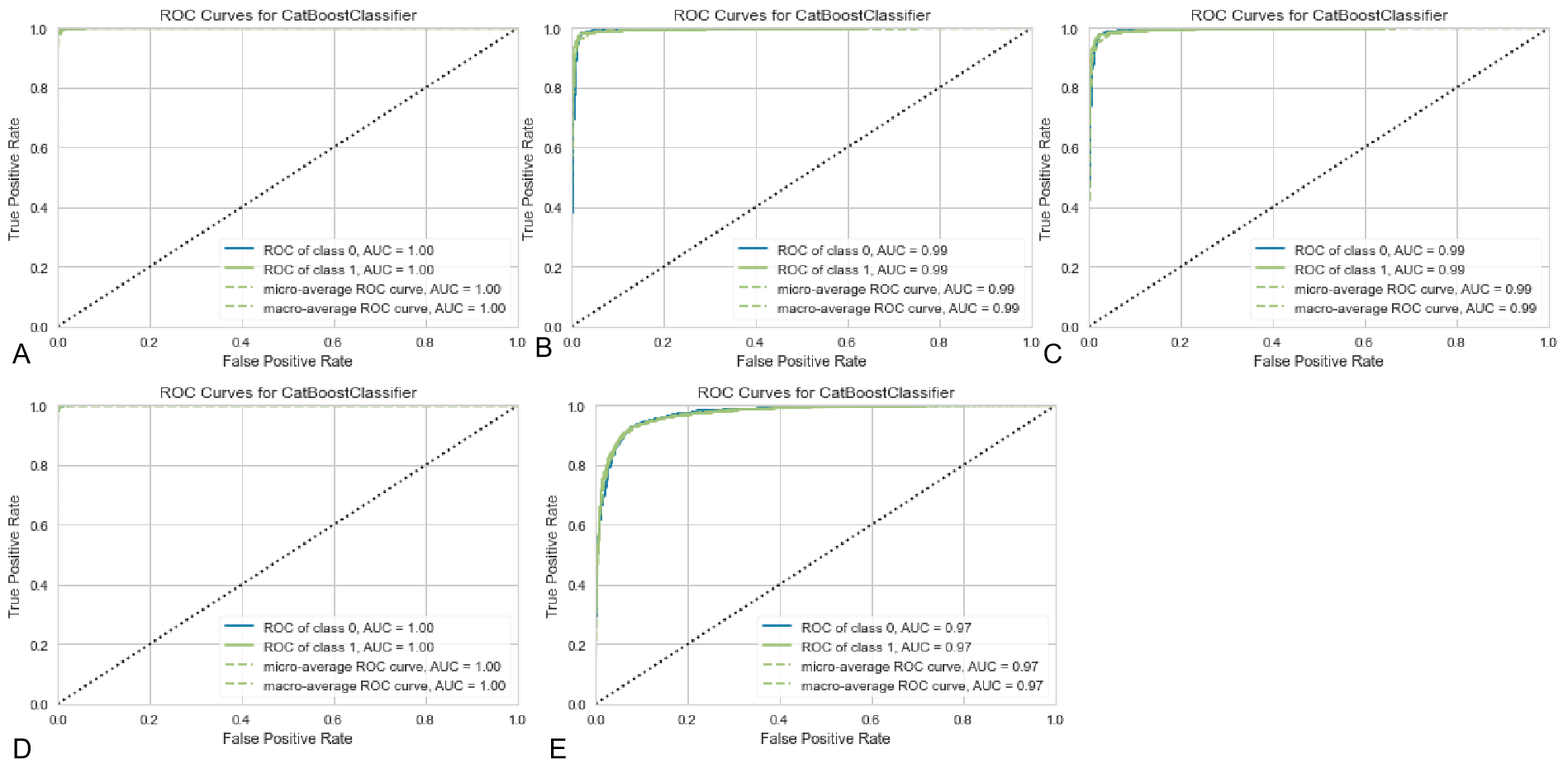


**Figure S3.** The CatBoost classifiers for predicting chlamydia(A), genital herpes(B), genital warts(C), gonorrhea(D), and overall STIs(E) based on the ROC plots in male populations.


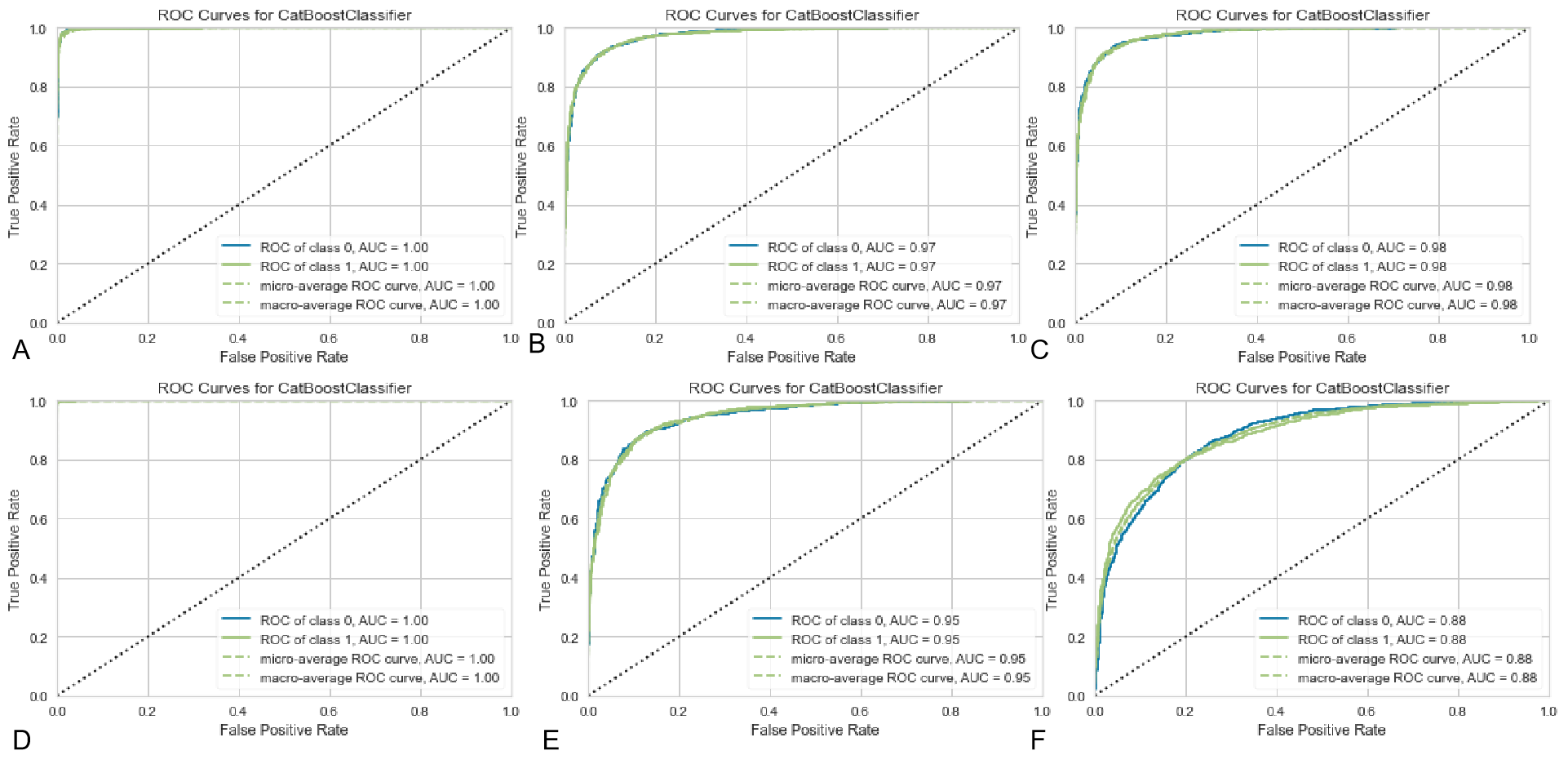


**Figure S4.** The CatBoost classifiers for predicting chlamydia(A), genital herpes(B), genital warts(C), gonorrhea(D), HPV(E) and overall STIs(F) based on the ROC plots in female populations.
